# Vehicle Target Detection of Autonomous Driving Vehicles in Foggy Environments Based on an Improved YOLOX Network

**DOI:** 10.3390/s25010194

**Published:** 2025-01-01

**Authors:** Zhaohui Liu, Huiru Zhang, Lifei Lin

**Affiliations:** College of Transportation, Shandong University of Science and Technology, Qingdao 266590, China; 202383160027@sdust.edu.cn (H.Z.); 17853118332@163.com (L.L.)

**Keywords:** foggy environment, visual sensors, vehicle target detection, attention mechanism, image enhancement, transfer learning

## Abstract

To address the problems that exist in the target detection of vehicle-mounted visual sensors in foggy environments, a vehicle target detection method based on an improved YOLOX network is proposed. Firstly, to address the issue of vehicle target feature loss in foggy traffic scene images, specific characteristics of fog-affected imagery are integrated into the network training process. This not only augments the training data but also improves the robustness of the network in foggy environments. Secondly, the YOLOX network is optimized by adding attention mechanisms and an image enhancement module to improve feature extraction and training. Additionally, by combining this with the characteristics of foggy environment images, the loss function is optimized to further improve the target detection performance of the network in foggy environments. Finally, transfer learning is applied during the training process, which not only accelerates network convergence and shortens the training time but also further improves the robustness of the network in different environments. Compared with YOLOv5, YOLOv7, and Faster R-CNN networks, the mAP of the improved network increased by 13.57%, 10.3%, and 9.74%, respectively. The results of the comparative experiments from different aspects illustrated that the proposed method significantly enhances the detection performance for vehicle targets in foggy environments.

## 1. Introduction

In recent years, the rapid evolution of computer technology and the advent of deep learning have spurred remarkable advancements in the field of image target detection. A plethora of high-performance target detection algorithms, predicated on convolutional neural networks (CNNs), have emerged. These algorithms can be broadly classified into two distinct categories: one-stage methods, represented by YOLOv1 [1] and SSD [2], and two-stage methods, represented by R-CNN [3], Fast R-CNN [4], and Faster R-CNN [5]. To ensure the operational safety of autonomous driving, target detection networks have to possess both a high accuracy and fast detection speeds. Balancing these requirements remains a significant challenge with the current methodologies. For example, one-stage models achieve faster detection speeds but often sacrifice accuracy, whereas two-stage models deliver superior accuracy at the expense of reduced detection speeds.

To bolster the precision of target detection, scholars have put forth a multitude of enhancements to the prevailing methodologies. Kumar [6] presented an enhanced YOLOv2 algorithm, developed using the joint version of ResNet and YOLOv2 to preform feature extraction and classification, respectively, which improved the accuracy in pedestrian detection. Xiao et al. [7] improved the Faster R-CNN algorithm with a skip pool and context information fusion. Wael [8] proposed a network parallelism approach that improves recognition accuracy and computational speed through algorithm fusion. Qiu et al. [9] introduced a vehicle recognition method that fuses edge features to enhance the model’s convergence speed and recognition accuracy.

To improve detection speed, researchers have focused on developing lightweight networks. Chen et al. [10] proposed DenseLightNet, a lightweight detection network that significantly reduces computational and storage demands for autonomous vehicles. It achieved detection speeds three times faster than that of YOLOv3 [11]. Junos et al. [12] introduced an optimized version of the YOLOv3-tiny network, named YOLO-P, which reduces the training speed and exhibits a high accuracy with an mAP of 98.68% and F1 score of 0.97. Xu et al. [13] reconstructed YOLOv3 and proposed a new lightweight target detection network, YOLOv3-Promote, which is five times faster than the original model and reduces the numbers of parameters by one-tenth.

Numerous scholarly investigations have addressed the constraints of target detection methodologies under optimal weather conditions, yet have largely overlooked the profound impact of adverse weather conditions, particularly the diminished visibility characteristic of foggy environments. Such conditions pose a significant challenge to both human drivers and sophisticated autonomous systems. Accurate environmental perception of visual sensors in foggy environments is crucial for ensuring safe driving; therefore, the improvement of target detection performance in such conditions has been highlighted as a research focus in the field.

Several studies have explored the impact of heavy fog on vehicular sensors and the accuracy of object detection within the driving environment. For instance, Ogunrinde et al. [14] analyzed the problem that the accuracy and performance of CNN-based target detectors often decline rapidly under bad weather conditions, and studied how to defog and restore the quality of fog images to improve the real-time performance of CNN-based target detectors. Liu et al. [15] quantitatively analyzed how visibility affects the detection accuracy of visual sensors under foggy conditions. The results revealed that as the fog density increased, the accuracy of object detection by the Faster R-CNN significantly decreased, with the detection accuracy dropping from 91.55% under clear weather conditions to 57.75% under heavy fog conditions.

A modest number of studies have investigated the repercussions of foggy weather on the fidelity of vehicle-mounted sensors and the precision of target detection in driving scenarios. Among these, several have delved into the application of image enhancement techniques to bolster target detection accuracy under such conditions. Image defogging has emerged as an essential methodology for enhancing detection accuracy. The existing image defogging algorithms can be classified into two main categories: enhancement algorithms based on image processing and image restoration methods of physical models. For instance, Liu et al. [16] introduced an improved Retinex defogging algorithm combining dark channel priors to enhance image details and colors for effective defogging. Chen et al. [17] introduced an improved Retinex algorithm to enhance the contrast of images captured in foggy environments. Lyes et al. [18] proposed a method that adjusts the parameters of the Dark Channel Prior (DCP) algorithm combined with the Fast Guided Filter, and quantifies the quality of the output images using various image quality metrics. Xie et al. [19] proposed an image defogging algorithm based on inverse channel compensation prior to addressing the serious halo artifacts and noise amplification problems in the image. Wang et al. [20] proposed a framework for small-object vehicle detection in hazy traffic environments (SHTDet), and designed an image enhancement (IE), whose parameters are predicted by a convolutional neural network [filter parameter estimation (FPE)].

Over the past few years, with the emergence of deep learning techniques, significant advancements have been made in the field of image dehazing. Yi et al. [21] introduced ClarifyNet, a novel, end-to-end trainable, convolutional neural network architecture for single-image dehazing. Liao [22] developed a fog removal algorithm based on a multi-scale parallel depth-separable convolutional neural network (MSP-DSCNN), which improved the quality of fog images and effectively solved the problems of color offset and poor visibility of details in the images. Furthermore, Song et al. [23] proposed a CPAD-Net defogging network model based on attention mechanism and dense residual blocks to achieve better defogging effects and restore more realistic and natural images.

Some studies have focused on enhancing target detection performance in foggy environments by improving network feature extraction methods. For instance, He et al. [24] developed an edge-guided dynamic feature fusion network (EDFFNet) to enhance the feature representation capability of the entire model in foggy weather. Wei et al. [25] combined Haar features and a Histogram of Oriented Gradients (HOG) to achieve better vehicle detection and tracking through a two-step process. Additionally, Singh et al. [26] proposed a bi-stream feature fusion (BFF) network for object detection in a hazy environment. The BFF network consists of three modules: a hybrid input, bi-stream feature extractor (BFE), and multi-level feature fusion modules.

Several studies have combined the aforementioned methods to further enhance target detection performance. For instance, Yu et al. [27] estimated atmospheric illumination and transmittance to defog images, subsequently using a convolutional neural network (CNN) for target detection. Hu et al. [28] proposed a joint semantic deep learning algorithm, which was constructed by embedding three concern modules and a 4-layer UNet multi-scale decoding module in the feature extraction module of the backbone network in Faster R-CNN to solve the problem of target detection in foggy road conditions. Additionally, Fang et al. [29] proposed an ODFC-YOLO model, which adopts a multi-task learning method for image defogging and object detection in foggy weather conditions.

Furthermore, several researchers have proposed alternative approaches to enhance the performance of detection networks in foggy environments. Hassaballah et al. [30] introduced a visibility image-processing enhancement method to address the challenge of low target detection accuracy in adverse weather conditions. Li et al. [31] proposed a union architecture BAD-Net that connects the dehazing module and detection module in an end-to-end manner. BAD-Net further improves detection performance through detection-friendly dehazing. Li et al. [32] proposed the integration of a hybrid convolutional module (HDC) into an all-in-one dehazing network, AOD-Net, to expand the perceptual domain for image feature extraction and refine the clarity of dehazed images. The ShuffleNetv2 lightweight network module has been incorporated into the backbone of the YOLOv5s network, and the Feature Pyramid Network (FPN) within the neck network has also been refined.

Despite the strides made in the field, state-of-the-art target detection methods under foggy weather conditions continue to face considerable challenges, which have been extensively documented in the scholarly literature. These challenges are multifaceted:

(1) The current target detection networks exhibit limited adaptability to varying densities of fog, resulting in elevated rates of miss detections and false alarms.
(2) The existing image defogging techniques are complex and time-consuming, which adversely impacts real-time performance in obstacle detection during the environment perception process.
(3) There is a notable scarcity of end-to-end approaches that effectively integrate image defogging with target detection. Additionally, current evaluation metrics for image defogging are insufficient, as they fail to account for the impact on target detection. This disconnect leads to poor coordination between defogging and detection processes, and it limits the overall performance enhancements.
(4) The available sample size of foggy image datasets of actual traffic scenes is frequently inadequate, necessitating the use of synthetic foggy images to bridge this gap. However, significant discrepancies between synthetic and real foggy images pose challenges in training robust deep learning models.

In response to the challenges inherent in the existing approaches, this study introduces an innovative vehicle target detection strategy tailored for foggy weather conditions, leveraging an enhanced YOLOX network (Exceeding YOLO Series in 2021). The proposed method exhibits several distinct advantages:

(1) This study focused on optimizing the target detection network itself, circumventing the requirement for intricate image defogging procedures, thereby enhancing detection efficacy while preserving real-time operational capabilities.
(2) The incorporation of attention mechanism modules into the network allows for the adaptive enhancement of target feature extraction in foggy images, significantly improving detection performance. Additionally, the use of an image data enhancement module addresses the scarcity of real foggy weather data, rendering the model more applicable in practical scenarios.
(3) Performance optimization was further achieved through the use of Focal Loss for the loss function, which considers the unique characteristics of foggy images and regulates the iterative calculation process, thereby enhancing model detection accuracy.
(4) Employing transfer learning techniques improves training efficiency and reduces training time. Training on a mixed dataset of clear and foggy environment images enables the network to absorb general as well as fog-specific features, yielding a more robust model.

## 2. Methodology

### 2.1. Introduction of YOLOX Network

**Figure 1:**
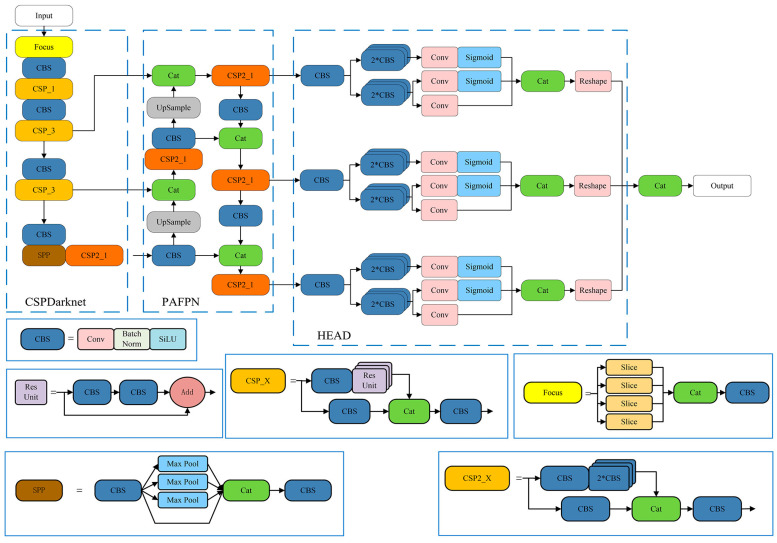
Diagram of YOLOX network structure.

The YOLO family of algorithms has achieved widespread acclaim in the field of target detection due to its remarkable performance. It is worth noting that YOLOX [33] introduces several significant modifications compared to its predecessors, enhancing its object detection capabilities. The architecture of the algorithm is divided into three main components: the backbone, the neck, and the prediction head. The structural diagram is presented in Figure 1.

The backbone component employs the CSPDarknet architecture, primarily tasked with extracting image features during the initial stage. The neck component adopts a PAFPN (Path Aggregation Feature Pyramid Network) structure, enhancing feature extraction by combining both upsampling and downsampling operations, and integrating features from various layers. The prediction component utilizes the YOLO head network structure, which is further divided into sub-components dedicated to regression and classification tasks. By integrating these components, the algorithm achieves the final detection outcomes.

### 2.2. Optimized Network Structure

#### 2.2.1. Attention Mechanism Module

The attention mechanism in deep learning draws inspiration from the attention model used by the human visual system, enabling the network to concentrate on pertinent information even when resources are constrained. This mechanism has proven to be indispensable across a spectrum of applications, including natural language processing (NLP) [34] and image recognition.

The application of convolutional neural networks (CNNs) in image processing requires the network to be capable of selectively focusing on informative content rather than the entire image. Integrating attention mechanisms into convolutional neural network architectures enables the network to adaptively concentrate on target objects, thereby enhancing its capability to extract relevant features from images.

In the field of deep learning, numerous attention mechanisms have been proposed, such as squeeze-and-excitation network (SE-Nets) [35], efficient channel attention networks (ECA-Nets) [36], and convolutional block attention module networks (CBAM-Nets) [37]. SE-Nets and ECA-Nets are classified as channel attention mechanisms, while CBAM-Nets integrate both channel and spatial attention mechanisms.

SE-Nets focus solely on channel attention by adaptively recalibrating feature responses based on inter-channel dependencies. Their two-step process includes a squeeze operation on the feature statistics and an excitation operation that generates channel weights.

ECA-Nets build on-Nets’ principles but emphasizes computational efficiency by bypassing the fully connected layer after pooling, replacing it with convolutional operations to capture local channel interactions. This allows ECA-Nets to be more efficient, though it may compromise their ability to model global relationships between channels.

CBAM-Nets distinguish themselves by integrating both channel and spatial attention mechanisms. They first enhance significant channels through channel attention and subsequently apply spatial attention to focus on relevant spatial features. However, this dual approach may be limited by the restrictions of the image resolution. This study evaluated these attention mechanisms through experimentation, comparing their detection results, and providing a comprehensive analysis.

**Figure 2:**
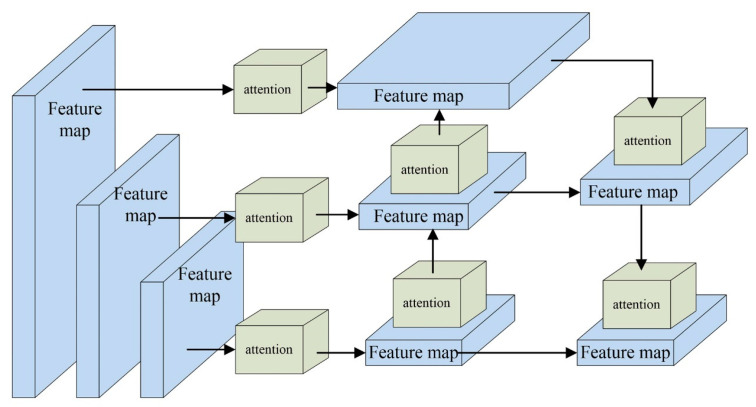
Working position of the attention mechanism module.

Foggy weather conditions reduce the contrast and brightness of images, leading to blurred edges and indistinct texture features, complicating the feature extraction process for networks. To address this issue, this study introduces an attention mechanism module to the YOLOX network. This feature extraction network utilizes the attention mechanism to learn the significance of each feature channel or spatial region, thereby enhancing its feature extraction capabilities and improving its adaptability to foggy environments. The attention mechanism module is strategically embedded at seven junctures throughout the network: three on the effective feature layer at the output of the backbone feature extraction network and four on the effective feature layer post-sampling in the enhanced feature extraction network. The placement of the attention mechanism module is illustrated in Figure 2.

#### 2.2.2. Image Data Enhancement Module

The application of image data augmentation techniques is crucial for enhancing the characteristics of the input data. This study aimed to expand the limited multi-fog image dataset and augment the features of vehicle targets within such images. In deep learning-based image object detection, the quality and size of the dataset play a pivotal role in the network’s performance. To address the issues of a limited sample size, poor image quality, and an uneven sample distribution in existing foggy image datasets, this research proposes an online image data augmentation technique to optimize the input data. The image data augmentation module is executed in real-time during the network training process, eliminating the need for separate preprocessing of image data and streamlining the operational procedure.

The image augmentation module in this study was designed to expand the dataset through various modifications, including random cropping and scaling, image flipping, and color space transformations. To guarantee the compatibility of the augmented images with the object detection network, the label annotations underwent automatic recalibration during the augmentation process. Through the implementation of these sophisticated techniques, the image data augmentation module successfully generated a diverse array of novel images for the model’s training regimen, thereby augmenting the richness of the image data’s characteristics. This methodology not only broadens the spectrum of training data diversity but also bolsters the model’s adaptability to fluctuations in object positioning and orientation, ultimately refining the network’s robustness and accuracy. As a result, this approach cultivates a more robust and versatile model, adept at navigating input data variations, and bolsters the performance in target detection tasks.

**Figure 3:**
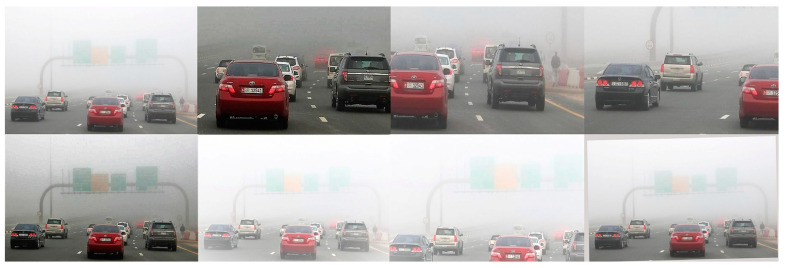
Effect of image data augmentation.

These modifications are designed to increase the volume of samples and enhance the quality and distribution of the samples, thereby enriching the dataset and strengthening the features. This approach aimed to improve the robustness of object detection networks under various foggy conditions. The effect of the image data enhancement is illustrated in Figure 3.

In addition to the online image data augmentation module, this study also utilized Mixup for image data augmentation during network training. The core concept of Mixup is to superimpose and blend two images to create a new one, which is then labeled with a combination of the original labels. Assuming there are two images, $x_{i}$ and $x_{j}$, with labels $y_{i}$ and $y_{j}$, respectively, the new image generated by Mixup can be represented by Equation (1):

(1) $$\hat{x}=\lambda x_{i}+(1-\lambda )x_{j}$$

where λ represents a parameter randomly sampled from a Beta distribution, which controls the proportion of the two original images in the generated new image. The label corresponding to the new image generated by Mixup can be represented by Equation (2):

(2) $$\hat{y}=\lambda y_{i}+(1-\lambda )y_{j}$$

**Figure 4:**
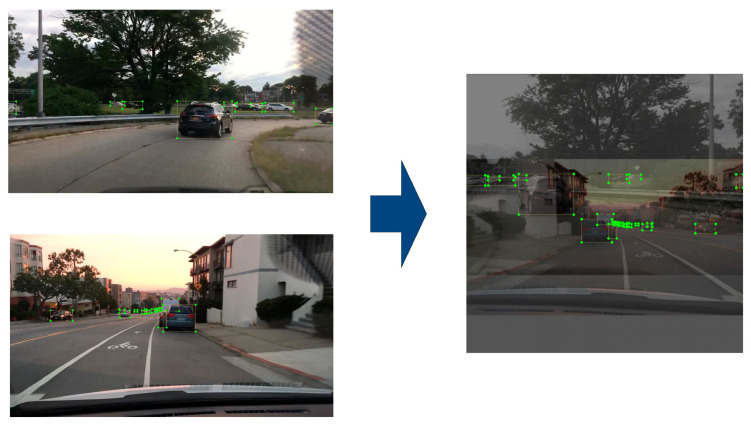
The effect of Mixup image data enhancement.

Mixup combines two images to produce a new image while simultaneously generating a corresponding target label, as illustrated in Figure 4. As observed in Figure 4, the color saturation of the newly generated image is reduced, and occlusion issues arise in the overlapping regions of the targets due to image superposition. These alterations are more akin to the characteristics of hazy images. Consequently, employing Mixup for image data augmentation can assist the model in learning features that are more indicative of hazy conditions, thereby enhancing the model’s robustness in such environments and facilitating the acquisition of more generalized features. This approach helps to mitigate the risk of overfitting. Furthermore, Mixup can expand the training dataset, thereby improving the model’s generalization capabilities.

### 2.3. Optimized Loss Function

YOLOX is a one-stage target detection network. Generally, the detection accuracy of one-stage networks is lower than that of two-stage networks, primarily due to the challenge of distinguishing between positive and negative samples caused by an imbalanced sample distribution. In foggy environments, the degradation of target features and the increased complexity of background features in images further exacerbate the difficulty of positive and negative sample classification. Additionally, an uneven fog distribution results in significant variability in the detection difficulty of different targets, exacerbating the weaknesses of YOLOX. To address these challenges, this study conducted an analysis of the YOLOX network’s loss function and proposed optimizations aimed at enhancing the network’s performance in foggy conditions.

The YOLOX loss function, comprising three components (localization loss, classification loss, and confidence loss), can be represented by Equation (3):

(3) $$Loss=Loss_{\mathrm{Reg}}+Loss_{Cls}+Loss_{Obj}$$

The confidence loss function in YOLOX utilizes a cross-entropy loss function, as illustrated by Equation (4):

(4) $$Loss_{Obj}=-y1by^{\prime }-(1-y)1(1-y^{\prime })=\left\{\begin{array}{cc} -1by^{\prime } & y=1\\ -1b(1-y^{\prime }) & y=0 \end{array}\right.$$

where y is the label and $y^{\prime }$ is the model output category probability.

When employing the YOLOX algorithm for target detection, the model generates a substantial number of candidate bounding boxes from which the final selections are made. Given the scarcity of vehicle targets within the images, a predominant portion of these candidate boxes typically represents the background rather than the vehicles of interest. In the realm of object detection, positive samples are defined as the actual targets intended for identification, whereas negative samples are largely indicative of the background. This disparity leads to a pronounced imbalance in the distribution between positive and negative samples, which can be detrimental to the model’s performance. The cross-entropy loss function, which is commonly utilized, assigns equal importance to each sample during the computation of loss. This equal weighting can hinder the model’s capacity to discern between positive and negative samples with precision, potentially impacting the accuracy of the detection process.

Moreover, the vehicle target features in the image are degraded by the foggy environment, while the background becomes more complex, exacerbating the imbalance between positive and negative samples. Under such conditions, the cross-entropy loss function often overemphasizes background feature information, hindering the effective learning of target features. In addition, the uneven distribution of fog in real-world environments causes varying degrees of target feature degradation, making accurate target detection increasingly difficult. This leads to suboptimal network training performance and detection outcomes, highlighting the need for loss function optimization to enhance the model’s robustness in foggy conditions. To address these issues, this study proposed optimizing the confidence loss function using Focal Loss [38], as demonstrated in Equation (5):

(5) $$L_{f}=\left\{\begin{array}{cc} -\alpha {\left(1-y^{\prime }\right)}^{\gamma }1by^{\prime }, & y=1\\ -(1-\alpha )y^{\prime }1b(1-y^{\prime }), & y=0 \end{array}\right.$$

where α and γ are parameters, α is a weighting factor, and γ is a modifiable factor.

In practice, the Focal Loss function serves as an enhancement of the cross-entropy function. To address the imbalance between positive and negative samples, the Focal Loss function assigns different weights to these samples through the α parameter. Positive samples, being fewer in number, are given higher weights, while negative samples are assigned lower weights due to their larger quantity. Additionally, to manage varying levels of difficulty in classifying samples, the Focal Loss function uses the γ parameter to assign different weights. It assigns larger weights to samples that are difficult to classify and smaller weights to those that are easier to classify. In this study, the values of the α and γ parameters were determined by referencing the literature [38] and through experimental validation.

### 2.4. Improved Network Structure

**Figure 5:**
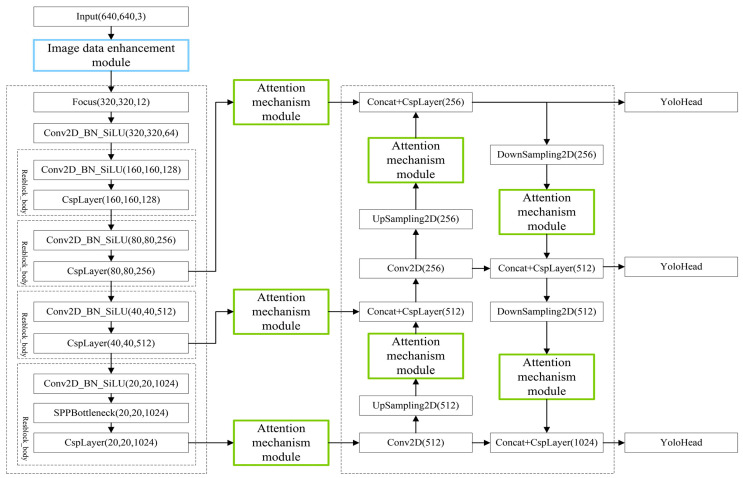
Improved overall network architecture.

This study enhanced the YOLOX network in two key aspects, as illustrated in Figure 5. Firstly, SE-Net, ECA-Net, and CBAM-Net attention mechanisms were integrated at specific points within the feature extraction network to improve target feature extraction in foggy images. The green boxes in the figure indicate the positions of these attention mechanisms, strategically placed at seven distinct points throughout the architecture. To evaluate the efficacy of these attention mechanism modules, three experiments were conducted. In the first experiment, SE-Nets were applied at the “Attention Mechanism Module” locations in Figure 5. In the second experiment, ECA-Nets were applied. Finally, in the third experiment, CBAM-Nets were employed. Additionally, an image data augmentation module was incorporated into the YOLOX target detection network for online data enhancement.

The network initiates the processing by resizing the input image to a uniform dimension of 640 × 640 pixels. Subsequently, the image enhancement module is used to augment the data. The CSPDarknet main feature extraction network is then employed to extract features, resulting in three effective feature layers. These layers are subsequently input into the PAFPN enhanced feature extraction network after passing through the attention mechanism module. Feature extraction and fusion are improved through upsampling and downsampling, producing three effective feature layers. Finally, these layers are input into the YOLO head, where regression and prediction are performed to accomplish the target detection task.

## 3. Experiments

### 3.1. Establishment of the Experimental Dataset

The real foggy image dataset RTTS has 4332 images and labels the categories of cars, buses, bicycles, motorcycles, and pedestrians, with over 40,000 labeling boxes. The details of the dataset can be found in Table 1.

However, due to the limited number of images in the RTTS dataset, it may not be sufficient for training a convolutional neural network to achieve satisfactory detection results. To address this issue, additional datasets such as the entire Foggy_Driving dataset and select images from the BDD100K dataset were incorporated. The final dataset used in this study contains 6000 images, all labeled using LabelImg 1.8. Given the study’s focus on vehicle target detection, the labels in the RTTS dataset were processed to include only vehicle labels. Due to the insufficient number of bus labels, it was challenging to meet the network training requirements, so only car labels were retained. In addition, the DAWN (Detection in Adverse Weather Nature) dataset consists of images of traffic scenes collected under various unfavorable weather conditions, and contains a total of 1000 images. These images are categorized into four groups based on the weather conditions: fog, snow, rain, and sandstorm. Among them, the DAWN-Fog dataset contains 300 foggy images, accompanied by corresponding label information.

The experimental training and validation set comprises images from the BDD100K, DAWN-Fog, and RTTS datasets, including both clear and foggy images, totaling 5820 images. The test set consisted of images from the Foggy_Driving dataset, the DAWN-Fog dataset, and the remaining images from the RTTS dataset. All images in the test set were real foggy images, totaling 780 images.

All experiments in this study were performed using the aforementioned dataset.

### 3.2. Model Training and Parameter Setting

The experimental hardware was AMD R7 CPU (AMD, Santa Clara, CA, USA) and NVIDIA 3060 GPU (AMD, Santa Clara, CA, USA). The experiment was carried out in the Pytorch 1.7 framework, using the Python 3.7 language.

**Figure 6:**
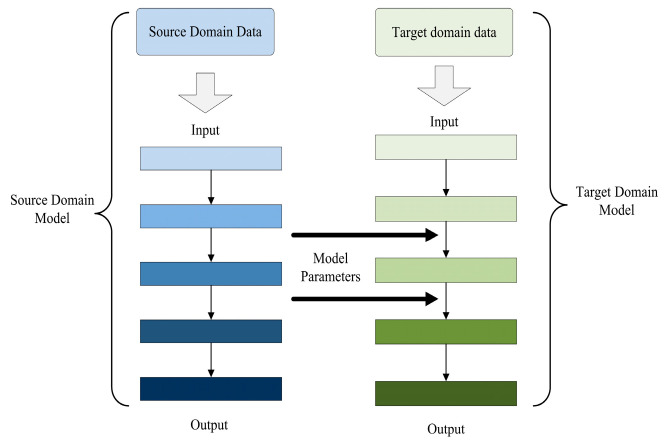
Schematic diagram of transfer learning.

In the application of deep learning for target detection in foggy environments, a significant challenge is the requirement for a large dataset. On the one hand, obtaining a sufficient quantity of real annotated foggy images is often quite challenging; on the other hand, the model training process itself demands a considerable time investment. To mitigate the aforementioned challenges, this research integrated transfer learning into the network training regimen. Transfer learning is a paradigm that leverages the knowledge encapsulated within a pre-trained model to expedite and enhance the training process of a novel model. This approach is particularly advantageous when dealing with complex environments, such as foggy conditions, where traditional training methods may falter. The spectrum of transfer learning strategies can be broadly classified into three distinct categories: sample-based, model parameter-based, and feature-based. In the context of this study, we opted for model parameter-based transfer learning. This method involves the direct application of pre-trained model parameters to the new model, thereby inheriting the learned features and patterns that are relevant to the task at hand, which entails sharing commonalities such as model parameters, prior knowledge, and architectures between the source and target tasks. The underlying principle of this approach is delineated in Figure 6.

In this study, we harnessed the power of transfer learning by acquiring the weights trained on the YOLOX network with the VOC (Visual Object Classes) dataset and integrating them into our enhanced network as initial pre-training weights. This strategic choice was motivated by two primary considerations. First and foremost, the incorporation of the attention mechanism module into the YOLOX network did not necessitate any modifications to the underlying backbone feature extraction network architecture. This preservation of structure ensures that the stability of the network is maintained upon the introduction of pre-trained weights. Secondly, the VOC dataset stands as a well-established benchmark for detection and recognition tasks, encompassing a diverse array of common targets encountered in autonomous driving scenarios, which bear a striking resemblance to the targets within the scope of our study. The adoption of this transfer learning approach yielded a dual benefit: it elevated the overall quality of our model and concurrently expedited the training process, achieving a substantial reduction in the time required for model convergence.

The loss function converged after 200 training epochs. During the initial 20 training epochs, the network was in a frozen state, wherein parameters of the backbone network remained unchanged while only the parameters of other components were adjusted. In this phase, the training of the network was relatively stable, and the hardware requirements were not stringent, allowing for a batch size of 32. In the subsequent 180 epochs, the network transitioned into a “thawing” phase, during which the parameters of the entire network were adjusted. This stage demanded a higher hardware performance, leading to a reduction in the batch size to 16. The maximum and minimum learning rates for the training process were set to 0.01 and 0.0001, respectively.

### 3.3. Experimental Results

#### 3.3.1. Experimental Results for Optimized Network Structure

(1) Experimental Results and Analysis of Optimized Image Data Augmentation Methods

To validate the effectiveness of the image data augmentation, the optimized method was tested on the test set both before and after augmentation, with the results shown in Table 2. By comparing and analyzing these test results, it was verified that the optimized image data augmentation method significantly improves the network’s performance in target detection under foggy conditions.

Based on the detection data presented in Table 2, Network 2 achieved a 3.5% increase in Recall, despite a slight decrease in Precision by 1.73%, while the mean Average Precision (mAP) improved by 1.76% compared to Network 1. Despite a minor reduction in Precision, the overall evaluation metric, mAP (mean Average Precision), showed an improvement, signifying a general improvement in the detection capabilities of the system. This enhancement can be attributed to the image data augmentation strategy implemented in this study, which was effective in bolstering the network’s performance in target detection under foggy conditions. The augmentation module integrated into the target detection network enriched the training dataset by introducing a more varied and extensive collection of sample data. This not only increased the dataset’s diversity but also fortified the network’s robustness, enabling it to generalize more effectively across different environmental conditions. Furthermore, the Mixup image data augmentation method increased the similarity of training set images to foggy images by blending them, which in turn improved the network’s adaptability to foggy environments.

(2) Experimental results and analysis of the imposed attention mechanism

Previous experiments have demonstrated that the optimized image data augmentation method can effectively improve the network’s target detection performance in foggy environments. Building on these findings, an attention mechanism module was added to the network to further optimize its target detection capabilities.

**Figure 7:**
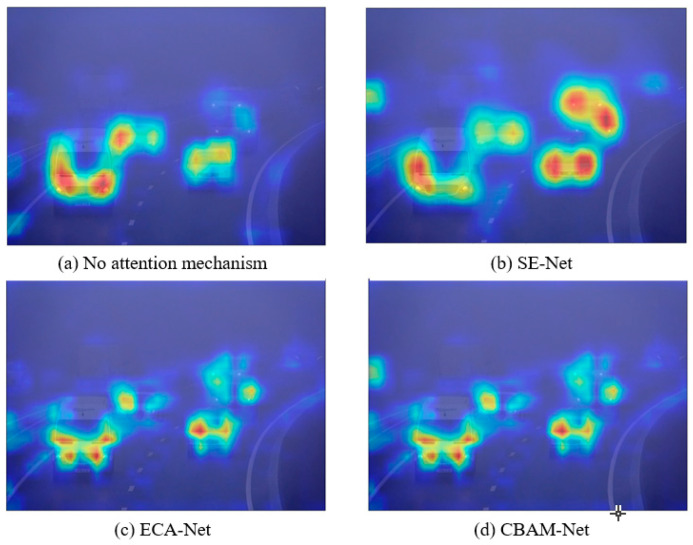
Grad-CAM heat map visualization results.

The attention mechanism module continuously adjusts the network’s weights in spatial or channel dimensions during the training process to emphasize the impact of important features on the model. This capability enables the network to adaptively enhance the extraction of target features within foggy images, subsequently improving target detection performance under foggy conditions. The experimental results demonstrated the significant role of the attention mechanism module in the network. However, describing the target detection process in deep learning is often challenging. To validate the role of the attention mechanism modules in the target detection network, a heat map visualization of the detected images was created using Grad-CAM. Figure 7 shows the heat maps generated by introducing the three different attention mechanism modules into the network for the images to be detected.

From the figure above, it can be observed that the introduction of the attention mechanism enhances the network’s focus on targets in the images. The four heat maps exhibited a more pronounced differentiation, indicating that the network is more adept at concentrating on closer targets, yet it tends to overlook those that are more distant. Figure 7 demonstrates that the network with the SE-Net attention mechanism module performed better in focusing on distant targets that are more affected by the foggy environment.

**Figure 8:**
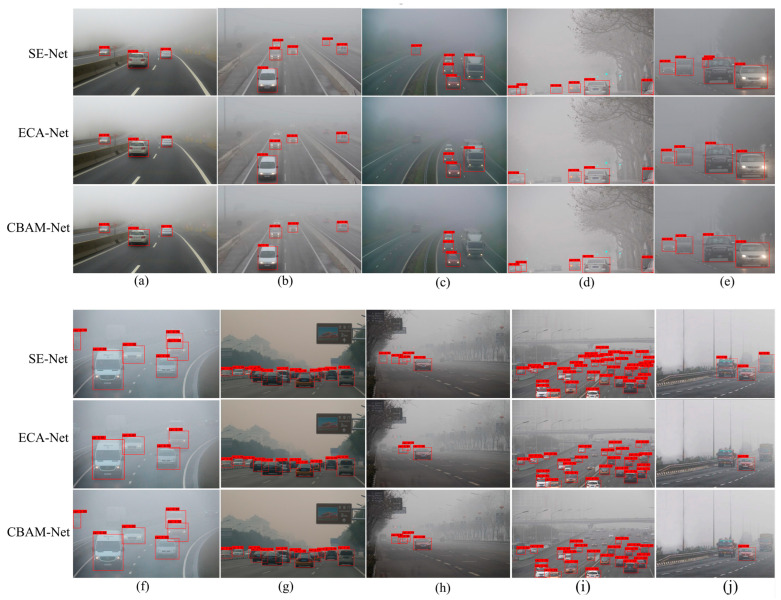
Detection effect of three different attention mechanism modules. (**a**)–(**j**) are the comparison of 10 sets of images randomly selected from the test results.

To provide a more intuitive visualization of the detection results, ten images from the detection results were selected for comparison, and the results using the different attention mechanism modules are shown in Figure 8.

Figure 8 demonstrates that the network with the SE-Net attention mechanism module detected the highest number of targets, experienced the fewest missed detections, and exhibited the greatest confidence levels for each identified target. This indicates that the SE-Net attention mechanism module plays the most significant role in improving target detection performance in foggy environments among the three attention mechanisms. The overall detection results can be roughly divided into three scenarios: ① Minimal Difference in Detected Targets: Figure 8a,g demonstrate only slight variances in the number of detected targets; however, notable differences in confidence levels for each target were evident. These figures depict targets that were closely situated and less impacted by the foggy conditions, resulting in minor differences in detection outcomes among the different models. ② Fewer Detected Vehicle Targets: In Figure 8j, fewer vehicle targets were detected, with few targets successfully detected. The models introducing ECA-Nets and CBAM-Nets only successfully detected one vehicle, while the model introducing SE-Nets detected three vehicles and missed one. The figure indicates that the vehicles on the left and right have unusual shapes and complex color patterns compared to the other vehicles in the image. ③ Pronounced Differences in Detection Results: The remaining images showed more pronounced differences in target detection, with the best results from the network incorporating the SE-Net attention mechanism module and poorer results from the networks with ECA-Nets and CBAM-Nets. These discrepancies were particularly evident in distant targets, where the foggy conditions considerably impaired the environmental visibility, thereby complicating the detection process.

To gain deeper insights into the effects of incorporating attention mechanism modules into the object detection network, this study performed ablation experiments. These experiments aimed to intuitively assess how the various attention mechanism modules influence detection performance in foggy environments. Table 3 shows the details of the network and the detection results.

As shown in Table 3, when the experimental dataset remained unchanged, the Recall, Precision, and mAP values of the improved networks increased to varying degrees, demonstrating the effectiveness of the network enhancements for foggy environments. Network 2, which builds upon Network 1 by incorporating an image data enhancement module, exhibited an 11.38% increase in mAP compared to Network 1. The image data enhancement module augments the original dataset by randomly transforming images, which provides the network model with a sufficient and diverse array of image target features for training. This ultimately leads to improved detection performance of the model.

Upon analyzing the visualization results, it can be concluded that introducing the attention mechanism module in the YOLOX target detection network significantly improved its performance in foggy environments. Among the three attention mechanisms evaluated, SE-Nets emerged as the most effective, enabling the network to better detect distant targets and those heavily influenced by fog. The SE-Net attention mechanism module also enhanced the YOLOX target detection network’s ability to detect targets with unique shapes and complex features.

SE-Nets and ECA-Nets are both channel attention mechanisms. Compared to SE-Nets, the ECA-Net attention mechanism reduces computation by eliminating the fully connected layer after global average pooling. However, this reduction in complexity comes at the cost of diminishing the effectiveness of the attention mechanism. In foggy environments, the complexity of target image features suggests that attention mechanisms that do not utilize fully connected layers after global average pooling do not perform as effectively as SE-Nets for vehicle target detection. CBAM-Nets combine channel and spatial attention mechanisms, but their spatial attention mechanism only partially applies to the spatial feature map and is limited by image resolution. Consequently, adding CBAM-Nets to the network does not result in better detection performance. Thus, it can be concluded that the SE-Net channel attention mechanism is more suitable for vehicle target detection in foggy environments.

#### 3.3.2. Experimental Results for Optimized Loss Function

To enhance the network’s detection performance, this study optimized the loss function of Network 3 using the Focal Loss function, and the resulting optimized network was denoted as Network 6. The parameters in the Focal Loss function were determined through a combination of referencing prior work [38] and experimental validation. This study conducted a comparison experiment for the values of the parameters γ and α in the Focal Loss function, and the experimental results are shown in Table 4. It can be seen from Table 4 that when α was 0.25 and γ was 2, the model had the highest mAP value, and the model had the best detection performance at this time.

**Figure 9:**
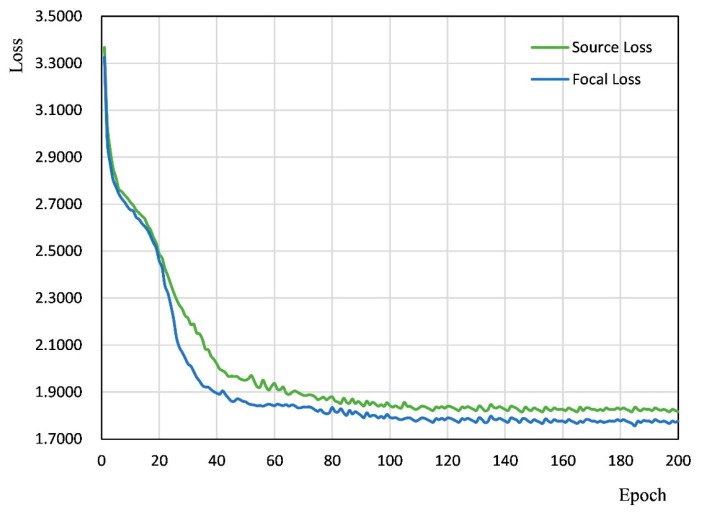
Comparison of loss function curves.

Figure 9 shows a comparison of the loss function curves before and after optimizing with Focal Loss. As depicted in Figure 9, the loss function curves exhibited significant differences post-optimization. Notably, during the early stages of network training, the differences between the two loss functions were relatively small, indicating that Focal Loss has a limited impact during the initial learning phase. However, after 20 epochs, the loss value decreased more rapidly when using Focal Loss, ultimately leading to convergence with a smaller loss value. This effect is clearly evident in the loss function graph. Focal Loss promoted faster convergence and enhanced loss values, which could have significant implications for a broad range of applications in deep learning.

**Figure 10:**
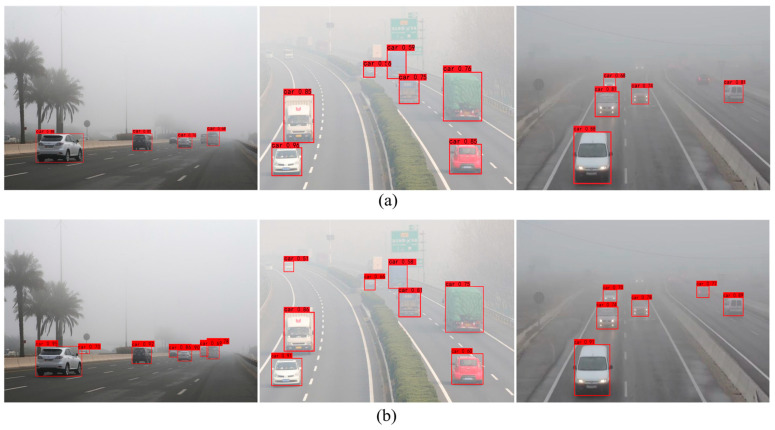
Comparison of visualization detection results before and after optimization of loss function of target detection network. (**a**) is the optimized loss function before, (**b**) is the optimized loss function after.

The visual detection results before and after optimizing the loss function are shown in Figure 10. From this figure, it is evident that the target detection network optimized with the new loss function showed an enhanced recognition capability for distant targets, helping to mitigate the issue of missed detections in foggy environments. Additionally, the detection boxes generated by the network for the same object had a higher confidence after optimizing the loss function. These results indicate that the proposed loss function optimization method can improve the detection performance of the network to a certain extent in foggy environments. By incorporating Focal Loss into the loss function of the YOLOX network model, the challenges associated with imbalanced positive and negative samples arising from the complex backgrounds in foggy images, as well as the significant variability in target detection difficulty due to different fog concentrations, can be effectively addressed. Consequently, the detection accuracy was notably improved while maintaining the detection speed.

Table 5 presents a comparative analysis of the detection results between Network 6 and Network 3. Network 6 demonstrated significant enhancements in Recall, Precision, and mean Average Precision (mAP) over Network 3. These improvements suggest that optimizing the network’s loss function with Focal Loss effectively boosted the performance of target detection in foggy conditions.

### 3.4. Comparison Experiments

The present study proposed a vehicle target detection method suitable for foggy environments, which consists of two parts: the first part is a target detection module based on an optimized YOLOX network, and the second part is an image style transfer module based on optimized CycleGAN. The performances of the target detection module and the style transfer module were analyzed and verified by setting up comparative experiments to prove the superiority of the proposed method. Since the image style transfer module aims to reduce the difference between foggy images and clear images, and its principle is similar to that of image defogging techniques, it was compared to the classical image defogging methods DehazeNet and GCANet to verify the foggy image processing performance of the style transfer module and its important role in target detection tasks in foggy environments. In addition, YOLO series target detection algorithms such as YOLOv5 [39] and YOLOv7 [40], as well as the classical two-stage target detection algorithm Faster R-CNN, were used for the comparative validation of the target detection module.

**Figure 11:**
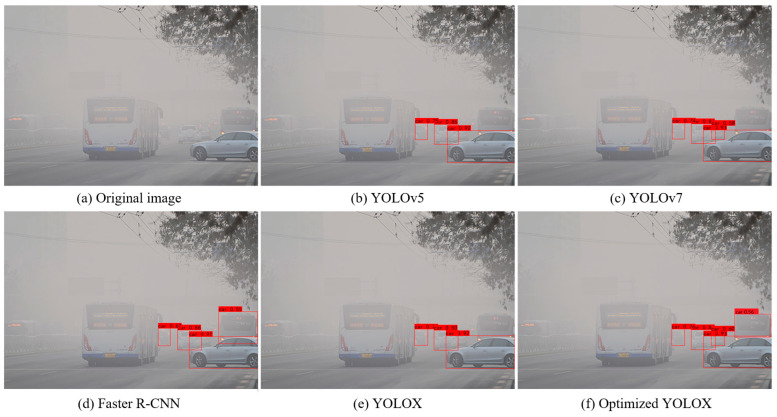
Visual comparison of performances of different object detection methods on foggy images. (**a**) is original image, (**b**) is YOLOv5, (**c**) is YOLOv7, (**d**) is Faster R-CNN, (**e**) is YOLOX, (**f**) is optimized YOLOX.

**Figure 12:**
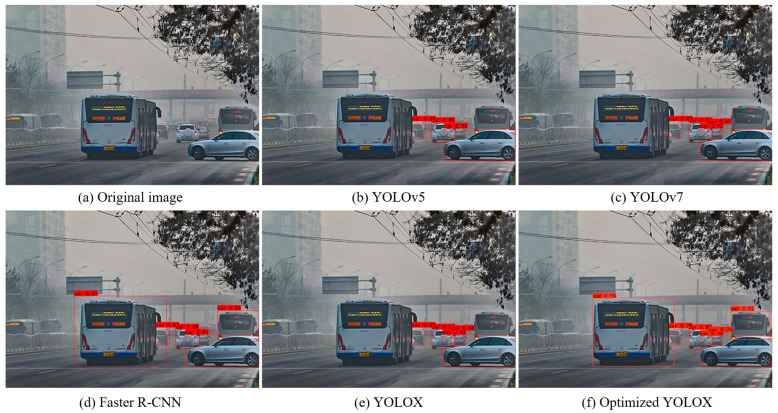
Visual comparison of performances of different object detection methods on style transfer images. (**a**) is original image, (**b**) is YOLOv5, (**c**) is YOLOv7, (**d**) is Faster R-CNN, (**e**) is YOLOX, (**f**) is optimized YOLOX.

Figure 11 and Figure 12 show the visual detection results of the different target detection methods for foggy images and style transferred images. As can be seen from the figures, in the original foggy images, all five target detection methods had more serious leakage problems, and the number of successfully detected vehicles were 3, 4, 4, 3 and 5, respectively. The optimized YOLOX network proposed in this paper achieved the best detection results. In the image after style transfer, the detection results of all five target detection methods were improved to some extent, and the number of successfully detected vehicles were 3, 4, 5, 4, 6, respectively, and the optimized YOLOX network still achieved the best detection results. The above analysis demonstrated that the proposed optimization scheme can effectively improve the detection performance of the target detection network, with the optimized network showing obvious advantages in the vehicle target detection task in both the original foggy images and the style-transferred images.

This study provides experimental results using the different detection methods on the test set to more accurately validate the effectiveness of the proposed network optimization scheme. The experimental results are summarized in Table 6. It can be concluded from the comparison of the experimental results that, when the original foggy image was detected directly without using the image processing module, the mAP of the optimized YOLOX network was significantly higher than that of the other target detection networks, and both its Recall and Precision values were higher. When the style transfer image-processing module was used, the optimized YOLOX network still achieved the highest mAP, with its detection performance markedly superior to that of the other networks. The experimental results demonstrate that the proposed network optimization scheme can effectively improve the detection performance of the target detection network, and the vehicle target detection performance of the optimized network outperformed that of YOLOv5, YOLOv7, Faster R-CNN, and the original YOLOX.

**Figure 13:**
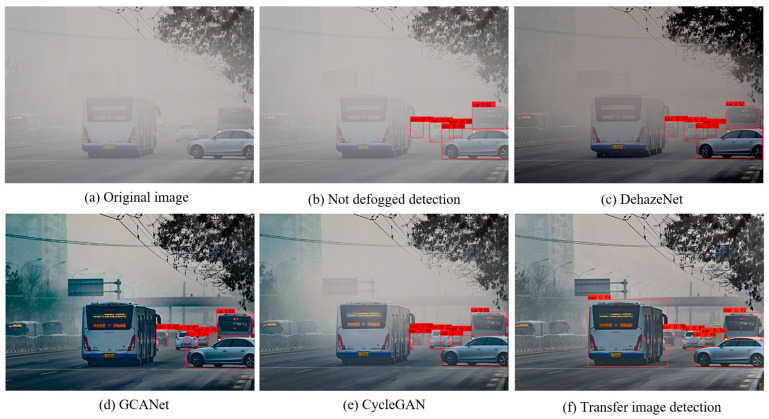
Comparative visualization of detection results for images using different methods. (**a**) is original image, (**b**) is not defogged detection, (**c**) is DehazeNet, (**d**) is GCANet, (**e**) is CycleGAN, (**f**) is transfer image detection.

Figure 13 illustrates the visual detection results of the optimized YOLOX network for images obtained using the different image processing methods. It can be seen that the vehicle targets in the original foggy image were missed more often, and the detection effect was improved to different degrees after processing the foggy image, with the image obtained after processing using the method exhibiting the best detection effect. The above analysis shows that the processing of foggy images can improve the target detection effect, with the style transfer method proposed in this paper demonstrating the most effective improvement.

Table 7 presents the detection results of the optimized YOLOX network on the images processed using the different methods. The data indicate that the use of image processing modules led to varying degrees of improvement in detection performance, primarily reflected in the enhancement of Recall values. Among these methods, the proposed image style transfer module achieved the best detection results, with an mAP of 88.99% and a Recall value of 81.25%, which are significantly higher than those of the other image processing methods. The improvement in image quality after processing reduces the difficulty of vehicle target recognition for the network, thereby enhancing the Recall value. The experimental results demonstrated that, compared to DehazeNet, GCANet, and CycleGAN, the proposed image style transfer module exhibits significant advantages in vehicle detection tasks under foggy conditions.

The visual detection results and experimental data demonstrated that the proposed target detection module significantly outperformed YOLOv5, YOLOv7, Faster R-CNN, and the original YOLOX algorithm in detection performance under foggy conditions. At the same time, the style transfer module, constructed using an optimized CycleGAN, exhibited superior image processing performance for foggy images compared to DehazeNet, GCANet, and the original CycleGAN-based methods. The results of the comparative experiments fully validate the superiority of the proposed vehicle detection method, which integrates YOLOX and style transfer, in accomplishing target detection tasks in foggy environments.

## 4. Conclusions

This study introduced an innovative approach for the detection of vehicle targets in foggy environments utilizing an enhanced YOLOX network. The development process involved several key steps: initially, input images underwent online image enhancement, which served to expand the foggy image dataset and enhance the network’s resilience in foggy conditions. Subsequently, attention mechanism modules, including SE-Net, ECA-Net, and CBAM-Net modules, were integrated at specific layers of the network to improve the extraction of target features from foggy images. Finally, the YOLOX loss function was optimized using Focal Loss, which significantly enhanced detection accuracy while preserving detection speed.

In this study, we initially visualized the impact of the three attention mechanism modules integrated into the YOLOX network using the Grad-CAM heat map visualization technique. The heat maps indicated that the SE-Net attention mechanism outperformed the ECA-Net and CBAM-Net mechanisms. The enhanced network was subsequently evaluated using real foggy images. The experimental results demonstrated that our improved target detection method significantly enhanced the Recall, Precision, and mAP values on the real foggy image test dataset compared to the original detection network. To further improve the network’s robustness, we incorporated an online image data augmentation module into the YOLOX network, effectively expanding the real foggy image dataset. The inclusion of attention mechanism modules allowed the network to adaptively focus on vehicle targets, which strengthened the convolutional neural network’s ability to extract target features in foggy conditions. Additionally, the Focal Loss function effectively addressed the challenges posed by imbalanced positive and negative samples in foggy environments, as well as the varying difficulty levels in sample classification. The improved network achieved Recall, Precision, and mAP values of 81.25%, 85.85%, and 88.99%, respectively. Compared to the original detection network, the overall detection performance of the improved network was significantly enhanced. The experimental results on the Foggy_Driving dataset demonstrated that the method proposed in this study offers substantial advantages for vehicle target detection in foggy environments.

Although our method performed excellently in terms of Precision and overall accuracy, particularly in vehicle detection tasks under foggy conditions, it is important to note that its Recall was relatively low. This suggests that while our approach has achieved some success in vehicle identification and localization, there is still room for further optimization in ensuring that all vehicle targets can be detected. The task of vehicle detection in dense fog environments is complicated by visual distortion, which makes it difficult for the detection system to accurately identify vehicles at long distances, thus adding to the complexity of the task. Moreover, achieving real-time detection is a key challenge that requires us to find the right balance between computational efficiency and model performance. In the future, our research direction will focus on improving the Recall of target detection networks. This could be achieved by adopting more advanced techniques, including an in-depth study of the network architecture, the development of more complex data augmentation strategies, and further optimization of the attention mechanisms. By overcoming these limitations, we aim to develop a target detection system that excels in all performance metrics, especially in challenging foggy environments, and ensure its applicability in real-time scenarios.

## Figures and Tables

**Table 1:** Information on the RTTS dataset.

Category	Car	Bus	Bicycle	Motorbike	Person
Number of label boxes	25,317	1136	2590	698	1232

**Table 2:** Object detection results before and after optimization of image data enhancement methods.

Network	Image Data Enhancement Module	Attention Mechanism Module	Recall	Precision	mAP
Network 1	-	-	62.44%	87.41%	74.69%
Network 2	√	-	65.94%	85.68%	76.45%

**Table 3:** Vehicle target detection results of different networks on a real foggy image dataset.

Network	Image Data Enhancement Module	Attention Mechanism Module	Recall	Precision	mAP
Network 2	√	-	65.94%	85.68%	76.45%
Network 3	√	SE	70.05%	92.72%	83.16%
Network 4	√	ECA	68.22%	88.73%	79.60%
Network 5	√	CBAM	68.61%	92.37%	81.88%

**Table 4:** Experimental results of parameter comparison.

Parameter	γ = 1	γ = 2	γ = 3
α = 0.25	84.95%	86.14%	79.59%
α = 0.50	84.64%	84.39%	81.75%
α = 0.75	82.22%	82.18%	82.36%

**Table 5:** Comparison of detection results before and after optimization of the network loss function.

Network	Image Data Enhancement Module	Attention Mechanism Module	Optimized Loss Function	Recall	Precision	mAP
Network 3	√	SE	-	70.05%	92.72%	83.16%
Network 6	√	SE	√	70.41%	94.35%	86.14%

**Table 6:** Detection results of different object detection methods.

Test	Image Processing Method	Target Detection Algorithm	Recall	Precision	mAP
1	-	YOLOv5	61.97%	88.01%	74.72%
2		YOLOv7	64.88%	90.69%	76.68%
3		Faster R-CNN	65.18%	90.15%	77.25%
4		YOLOX	62.44%	87.41%	74.69%
5		Optimized YOLOX	70.41%	94.35%	86.14%
6	Image Style Migration Module	YOLOv5	62.93%	87.79%	75.42%
7		YOLOv7	67.59%	90.75%	78.69%
8		Faster R-CNN	66.73%	90.20%	79.25%
9		YOLOX	65.83%	90.19%	77.58%
10		Optimized YOLOX	81.25%	85.85%	88.99%

**Table 7:** Detection results of optimized YOLOX network for images obtained using different methods.

Test	Target Detection Algorithm	Image Processing Method	Recall	Precision	mAP
1	Optimized YOLOX	-	70.41%	94.35%	86.14%
2		DehazeNet	72.11%	89.86%	85.62%
3		GCANet	75.37%	90.18%	87.14%
4		CycleGAN	74.40%	92.79%	86.55%
5		Image Style Migration Module	81.25%	85.85%	88.99%

## Data Availability

The data that support the findings of this study are available from the corresponding author upon reasonable request.

## References

[1] Redmon J., Divvala S.K., Girshick R.B., Farhadi A. You only look once: Unified, real-time object detection. Proceedings of the IEEE Conference on Computer Vision and Pattern Recognition.

[2] Liu W., Anguelov D., Erhan D., Szegedy C., Reed S., Fu C.-Y., Berg C. Ssd: Single shot multibox detector. Proceedings of the Computer Vision–ECCV 2016: 14th European Conference.

[3] Girshick R., Donahue J., Darrell T., Malik J. (2015). Region-based convolutional networks for accurate object detection and segmentation. IEEE Trans. Pattern Anal. Mach. Intell..

[4] Girshick R. (2015). Fast r-cnn. arXiv.

[5] Ren S., He K., Girshick R., Sun J. (2016). Faster r-cnn: Towards real-time object detection with region proposal networks. IEEE Trans. Pattern Anal. Mach. Intell..

[6] Geethapriya S., Kumar P. (2020). An Improved Pedestrian Detection Algorithm using Integration of Resnet and Yolo V2. Int. J. Recent Technol. Eng..

[7] Xiao Y., Wang X., Zhang P., Meng F., Shao F. (2020). Object detection based on faster R-CNN algorithm with skip pooling and fusion of contextual information. Sensors.

[8] Wael F. (2020). A comprehensive vehicle-detection-and-tracking technique for autonomous driving. Int. J. Comput. Digit. Syst..

[9] Qiu L., Zhang D., Tian Y., Al-Nabhan N. (2021). Deep learning-based algorithm for vehicle detection in intelligent transportation systems. J. Supercomput..

[10] Chen L., Ding Q., Zou Q., Chen Z., Li L. (2020). DenseLightNet: A light-weight vehicle detection network for autonomous driving. IEEE Trans. Ind. Electron..

[11] Redmon J. (2018). Yolov3: An incremental improvement. arXiv.

[12] Junos M.H., Mohd Khairuddin A.S., Thannirmalai S., Dahari M. (2021). An optimized YOLO-based object detection model for crop harvesting system. IET Image Process..

[13] Xu H., Guo M., Nedjah N., Zhang J., Li P. (2022). Vehicle and pedestrian detection algorithm based on lightweight YOLOv3-promote and semi-precision acceleration. IEEE Trans. Intell. Transp. Syst..

[14] Ogunrinde I., Bernadin S. (2021). A review of the impacts of defogging on deep learning-based object detectors in self-driving cars. SoutheastCon.

[15] Liu Z., He Y., Wang C., Song R. (2020). Analysis of the influence of foggy weather environment on the detection effect of machine vision obstacles. Sensors.

[16] Liu X., Liu C., Lan H., Xie L. (2020). Dehaze enhancement algorithm based on retinex theory for aerial images combined with dark channel. Open Access Libr. J..

[17] Chen Z., Wang L., Wang C., Zheng Y. Fog image enhancement algorithm based on improved Retinex algorithm. Proceedings of the 2022 3rd International Conference on Electronic Communication and Artificial Intelligence (IWECAI).

[18] Aksas L., Lapray P.-J., Foulonneau A., Bigué L. Joint qualitative and quantitative evaluation of fast image dehazing based on dark channel prior. Proceedings of the Unconventional Optical Imaging III, SPIE.

[19] Xie S., Zhang J. Image dehazing algorithm based on color correction and channel compensation prior. Proceedings of the 4th International Conference on Information Science, Electrical, and Automation Engineering (ISEAE 2022).

[20] Wang Y., Qin Y., Jiang H., Lu Y. (2023). Small object detection for autonomous driving under hazy conditions on mountain motorways. Opt. Eng..

[21] Yi W., Dong L., Liu M., Zhao Y., Hui M., Kong L. (2022). DCNet: Dual-cascade network for single image dehazing. Neural Comput. Appl..

[22] Liao J. (2022). Optimization and Application of Image Defogging Algorithm Based on Deep Learning Network. Sensors.

[23] Song C., Liu J. (2023). A Single Image Dehazing Method Based on End-to-End CPAD-Net Network in Deep Learning Environment. J. Circuits Syst. Comput..

[24] He W., Guo J., Wang Y., Zheng S. (2023). Edge-guided dynamic feature fusion network for object detection under foggy conditions. Signal Image Video Process..

[25] Wei Y., Tian Q., Guo J., Huang W., Cao J. (2019). Multi-vehicle detection algorithm through combining Harr and HOG features. Math. Comput. Simul..

[26] Singh K., Parihar A.S. (2024). Bff: Bi-stream feature fusion for object detection in hazy environment. Signal Image Video Process..

[27] Yu G., Wang S., Li M., Guo Y., Wang Z. (2019). Vision-based vehicle detection in foggy days by convolutional neural network. Proceedings of the 2019 Chinese Intelligent Systems Conference: Volume III 15th.

[28] Hu M., Li Y., Fan J., Jing B. (2022). Joint Semantic Deep Learning Algorithm for Object Detection under Foggy Road Conditions. Mathematics.

[29] Fang W., Zhang G., Zheng Y., Chen Y. (2023). Multi-Task Learning for UAV Aerial Object Detection in Foggy Weather Condition. Remote Sens..

[30] Hassaballah M., Kenk M.A., Muhammad K., Minaee S. (2020). Vehicle detection and tracking in adverse weather using a deep learning framework. IEEE Trans. Intell. Transp. Syst..

[31] Li C., Zhou H., Liu Y., Yang C., Xie Y., Li Z., Zhu L. (2023). Detection-friendly dehazing: Object detection in real-world hazy scenes. IEEE Trans. Pattern Anal. Mach. Intell..

[32] Li A., Xu G., Yue W., Xu C., Gong C., Cao J. (2024). Object Detection in Hazy Environments, Based on an All-in-One Dehazing Network and the YOLOv5 Algorithm. Electronics.

[33] Ge Z. (2021). Yolox: Exceeding yolo series in 2021. arXiv.

[34] Hu D. (2020). An introductory survey on attention mechanisms in NLP problems. Proceedings of the Intelligent Systems and Applications: Proceedings of the 2019 Intelligent Systems Conference (IntelliSys).

[35] Hu J., Shen L., Sun G. Squeeze-and-excitation networks. Proceedings of the IEEE Conference on Computer Vision and Pattern Recognition.

[36] Wang Q., Wu B., Zhu P., Li P., Zuo W., Hu Q. ECA-Net: Efficient channel attention for deep convolutional neural networks. Proceedings of the IEEE/CVF Conference on Computer Vision and Pattern Recognition.

[37] Woo S., Park J., Lee J.-Y., Kweon I.S. Cbam: Convolutional block attention module. Proceedings of the European Conference on Computer Vision (ECCV).

[38] Lin T. (2017). Focal loss for dense object detection. arXiv.

[39] Zhu X., Lyu S., Wang X., Zhao Q. TPH-YOLOv5: Improved YOLOv5 based on transformer prediction head for object detection on drone-captured scenarios. Proceedings of the IEEE/CVF International Conference on Computer Vision.

[40] Wang C.-Y., Bochkovskiy A., Liao H.-Y.M. YOLOv7: Trainable bag-of-freebies sets new state-of-the-art for real-time object detectors. Proceedings of the IEEE/CVF Conference on Computer Vision and Pattern Recognition.

